# The Stegomyia Mosquito

**Published:** 1904-05

**Authors:** L. O. Howard

**Affiliations:** San Antonio, Texas


### Correspondence.

### The Stegomyia Mosquito.

San Antonio, Texas, April 19, 1904.

*Editor Texas Medical Journal:*

In your issue of this month, on pages 418,419, Dr. Graves has utterly misrepresented the meaning of my paper in the United States Weekly Health Report in regard to the geographic distribution of the yellow fever mosquito. He has quoted the list of localities from which, prior to last November, I had actually received specimens of this mosquito, and from the absence of San Antonio on the list he has disingenuously made the inference that *Stegomyia* does not occur in this city. As a matter of fact in the rest of the paper, from which he does not quote, I show that the yellow fever mosquito must occur in almost every city in Texas, as well as in all of the other Gulf States.

I beg that you will explain this, as I do not wish to appear in the eyes of your readers quite as ignorant as Dr. Graves would seem to wish to make me. I trust that you will see the justice of such action.

Very truly,

(Signed) L. O. Howard.

[Dr. Howard is the entomologist of the United States Agricultural Department.—Ed.]

